# Neurotrophin‐3 stimulates stem Leydig cell proliferation during regeneration in rats

**DOI:** 10.1111/jcmm.15886

**Published:** 2020-10-22

**Authors:** Yige Yu, Zengqiang Li, Feifei Ma, Quanxu Chen, Liben Lin, Qiang Xu, Yang Li, Xiu Xin, Peipei Pan, Tongliang Huang, Yiyan Wang, Qianjin Fei, Ren‐Shan Ge

**Affiliations:** ^1^ Department of Obstetrics and Gynecology The Second Affiliated Hospital and Yuying Children's Hospital of Wenzhou Medical University Wenzhou China; ^2^ Department of Anesthesiology The Second Affiliated Hospital and Yuying Children's Hospital of Wenzhou Medical University Wenzhou China; ^3^ Reproductive Medicine Center The First Affiliated Hospital of Wenzhou Medical University Wenzhou China

**Keywords:** differentiation, NT‐3, proliferation, stem Leydig cell, Trkc

## Abstract

Neurotrophin‐3 (NT‐3) acts as an important growth factor to stimulate and control tissue development. The NT‐3 receptor, TRKC, is expressed in rat testis. Its function in regulation of stem Leydig cell development and its underlying mechanism remain unknown. Here, we reported the role of NT‐3 to regulate stem Leydig cell development in vivo and in vitro. Ethane dimethane sulphonate was used to kill all Leydig cells in adult testis, and NT‐3 (10 and 100 ng/testis) was injected intratesticularly from the 14th day after ethane dimethane sulphonate injection for 14 days. NT‐3 significantly reduced serum testosterone levels at doses of 10 and 100 ng/testis without affecting serum luteinizing hormone and follicle‐stimulating hormone levels. NT‐3 increased CYP11A1‐positive Leydig cell number at 100 ng/testis and lowered Leydig cell size and cytoplasmic size at doses of 10 and 100 ng/testis. After adjustment by the Leydig cell number, NT‐3 significantly down‐regulated the expression of Leydig cell genes (*Lhcgr, Scarb1, Star, Cyp11a1, Hsd3b1, Cyp17a1, Hsd17b3, Hsd11b1, Insl3, Trkc* and *Nr5a1*) and the proteins. NT‐3 increased the phosphorylation of AKT1 and mTOR, decreased the phosphorylation of 4EBP, thereby increasing ATP5O. In vitro study showed that NT‐3 dose‐dependently stimulated EdU incorporation into stem Leydig cells and inhibited stem Leydig cell differentiation into Leydig cells, thus leading to lower medium testosterone levels and lower expression of *Lhcgr, Scarb1, Trkc* and *Nr5a1* and their protein levels. NT‐3 antagonist Celitinib can antagonize NT‐3 action in vitro. In conclusion, the present study demonstrates that NT‐3 stimulates stem Leydig cell proliferation but blocks the differentiation via TRKC receptor.

## 1 INTRODUCTION

In mature male mammals, testicular adult Leydig cells (ALCs) are cells that primarily produce testosterone (T).^1^ During the postnatal development, ALCs are differentiated from stem Leydig cells (SLCs).^2^ Between ALCs and SLCs, there are two immediate stages, called progenitor Leydig cell (PLCs) and immature Leydig cells (ILC).^3^ Previous studies have shown that ethane dimethyl sulphone (EDS) can accurately kill all ALCs in rat testis, and then start to regenerate ALCs.^4^, ^5^ On the 21st day after EDS, development of PLCs is shown by the expression of the Leydig cell lineage biomarkers: lutropin‐choriogonadotropic hormone receptor (LHCGR), high‐density lipoprotein receptor (SCARB1), steroidogenic acute regulatory protein (STAR), cholesterol side‐chain cleavage enzyme (CYP11A1), 3β‐hydroxysteroid dehydrogenase/Δ(5)‐Δ(4) isomerase type I (HSD3B1), cytochrome 17α‐hydroxylase/17,20 lyase/17,20 desmolase (CYP17A1) and steroid 5α‐reductase type I (SRD5A1).^6^ At this stage, PLCs are unable to produce testosterone due to the lack of 17β‐hydroxysteroid dehydrogenase type 3 (HSD17B3) enzyme catalysis, but they synthesize androsterone.^6^ Twenty‐eight days after EDS, the next advanced ILCs appear.^6^ To judge that the PLC enters the ILC stage, two biomarkers, HSD17B3 and 11β‐hydroxysteroid dehydrogenase type 1 (HSD11B1), a glucocorticoid metabolic enzyme, appear in ILCs.^6^ Fifty‐six days after EDS, ALCs are fully regenerated and the ability to produce T has also been completely restored.^6^

Although several growth factors such as platelet‐derived growth factors^7^ and desert hedgehog^8^ and cytokines (interleukin‐6^9^ and oncostatin M^10^) have been identified to regulate the regeneration of ALCs (see review 2), other factors may also determine the development of SLCs into the Leydig cell lineage. Some studies have shown that some nerve growth factors such as nerve growth factor (NGF) may also be critical for the development of ALCs.^11^ Here, we described another neurotrophin, neurotrophin‐3 (NT‐3). NT‐3 is the third member in the NGF series of neurotrophins. NT‐3 has been shown to support the survival and differentiation of neurons.^12^ Interestingly, NT‐3 is also expressed in the mammalian testis.^13^ NT‐3 binds to the receptor TRKC, which is a member of the trk family of tyrosine protein kinase.^14^ Before birth, NT‐3 is involved in regulating the formation of seminiferous cords and germ cell differentiation, and in the determination of male gender.^14^ NT‐3 is secreted by Sertoli cells in the testis during the embryonic development.^14^ NT‐3 protomor contains the binding sites of Sertoli cell transcription factor, SOX9 and SOX9 stimulates the expression of NT‐3.^15^ In 13‐14 embryonic days, large amounts of NT‐3 are produced in the mouse testis and the knock out of NT‐3 and TRKC can reduce testicular interstitium size.^16^ This indicates that NT‐3 regulates the development of Leydig cells. Previous studies have demonstrated that SLCs are mainly distributed on the surface of seminiferous tubules (STs) in rat testes.^17^ SLCs can develop into ALCs under the induction of Leydig cell differentiation medium (LDM), which contains insulin‐transferrin‐selenium, luteinizing hormone (LH) and lithium ions.^17^, ^18^ In the current study, we used an in vivo EDS‐treated ALC regeneration model and an in vitro SLC culture to study the role of NT‐3 in SLC development.

## 2 MATERIALS AND METHOD

### 2.1 Chemicals and kits

Details of materials and methods are contained in Supporting information S1. Chemicals, test kits, equipment and software are included in Supporting information S2. The primers used for gene expression are included in Supporting information S3. Supporting information S4 contains antibodies for immunohistochemical staining and Western blotting.

### 2.2 Animal study for EDS‐treated SLC regeneration

Twenty four male Sprague Dawley rats were transported to Wenzhou Medical University and adapted for a week. ALCs were eliminated using 75 mg/kg EDS as previously described.^10^ Male rats were then randomly divided into three groups, eight in each group. From the 14th to the 28th day after EDS administration, we injected 0 (normal saline), 10 or 100 ng/testis NT‐3 into each testis. Intratesticular injection of NT‐3 was chosen to avoid the systemic effects of NT‐3 on the hypothalamic‐pituitary‐gonadal axis. On the 14th day after NT‐3 treatment, rats were euthanized with carbon dioxide, and blood and testis were collected. The animal experiment protocol was approved by the Animal Protection and Use Committee of Wenzhou Medical University.

### 2.3 Determination of serum and medium T

Both serum and medium T concentration was detected by Immulite2000 Total Testosterone kit as previously mentioned.^10^ Normal male rat serum (2 ng/mL) was used as internal quality control. The minimum determine concentration of T is 0.2 ng/mL.

### 2.4 ELISA for serum LH and FSH Levels

Luteinizing hormone and FSH levels were assayed using ELISA kits as described previously.^10^ After sample reacts with peroxidase‐conjugated IgG anti‐LH or anti‐FSH, substrate was added and plate was read.

### 2.5 qPCR

A testicle each rat was picked and put in Trizol solution, total RNAs were extracted, cDNA was generated, qPCR was performed as previously mentioned.^10^ Following mRNAs: *Lhcgr*, *Scarb1*, *Star*, *Cyp11a1*, *Hsd3b1*, *Cyp17a1*, *Hsd17b3*, *Insl3*, *Hsd11b1, Trkc*, *Nr5a1*, *Sox9* and *Rps16* were detected. The target mRNA level was adjusted to *Rps16*.

### 2.6 Western blot analysis

The Western blot technique was selected as previously described.^10^ Following primary antibodies, ACTB, LHCGR, SCARB1, STAR, HSD11B1, NR5A1, phosphorylated AKT1 (pAKT1), AKT1, phosphorylated mTOR (pmTOR), mTOR, phosphorylated 4EBP‐1 (p4EBP), 4EBP and ATP5O, were used. The data of the target protein was normalized to ACTB.

### 2.7 Preparation of testis tissue array for immunohistochemical staining and stereological counting of cells

A tissue array was prepared as described previously.^10^ The tissue‐array block was cut into 5 μm thick sections.

### 2.8 Immunohistochemistry and immunofluorescence staining of testis

According to the previously reported method,^10^ Immunohistochemical staining was performed. CYP11A1 (a biomarker for the Leydig cell lineage) or HSD11B1 (a biomarker of Leydig cells at the ILC and ALC stages) were used.

### 2.9 Calculation of Leydig cell and Sertoli cell number per testis

In order to count CYP11A1‐positive or HSD11B1‐positive Leydig cells or SOX9‐positive Sertoli cells, the fractionator technique was used for the above tissue‐array section as described.^10^

### 2.10 ST culture for SLC developmental assay

To investigate whether NT‐3 can affect the development of SLCs, an in vitro culture system of SLCs on the surface of STs was used as previously mentioned.^10^ STs were cultured with various concentrations of NT‐3 with or without antagonist Celitinib. Medium T concentrations were determined as above.

### 2.11 Incorporation of EdU into SLCs

As mentioned earlier,^10^ the incorporation of EdU into SLCs is measured by EdU Alaxa Fluor kit. EdU‐positive cells were counted.

### 2.12 Statistical analysis

The data are presented using the mean ± SEM *P* < .05 was considered statistically significant. One‐way ANOVA then Sidak‐adjusted Dunnett multiple comparison test or paired student *t* test (only for Western blotting analysis) was used to compare them with controls.

## 3 RESULTS

### 3.1 NT‐3 inhibits testosterone secretion in vivo

**Figure 1:**
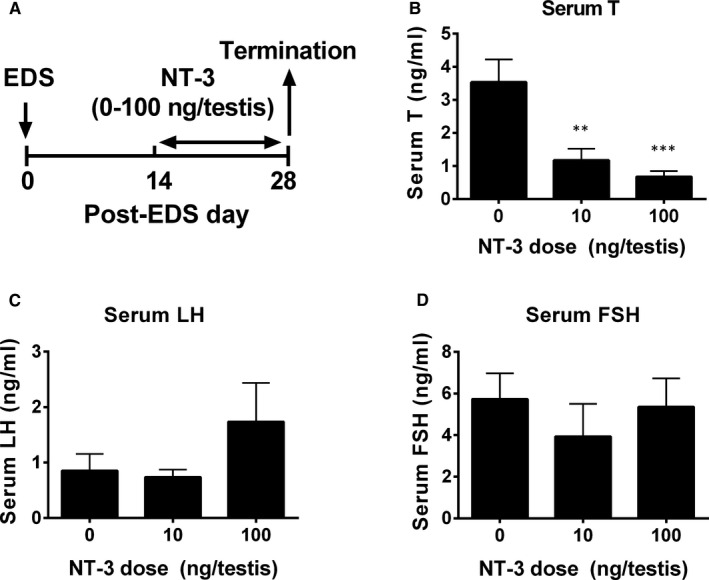
NT‐3 experimental protocol and serum testosterone (T), LH and FSH levels after in vivo NT‐3 treatment. (A) Experimental protocol; (B‐D) Serum T, LH and FSH levels. Mean ± SEM, n = 8. Asterisks (**, ***) designate significant differences from the control (NT‐3, 0 ng/ testis) at *P* < .01 and 0.001, respectively

In order to study the effect of NT‐3 on the development of SLCs, we used the EDS model to eliminate ALCs. NT‐3 (0, 10 or 100 ng/testis/d) was administered to rats by intratesticular injection for 14 days (Figure 1A). At the end of the treatment, NT‐3 did not affect the weights of the body and testes and relative testes (divided by body weight) when compared with controls (Supporting information S5). NT‐3 significantly inhibited serum T levels at doses of 10 and 100 ng/testis (Figure 1B). However, NT‐3 did not change serum LH (Figure 1C) and FSH (Figure 1D) levels. These data indicate that NT‐3 exerts its effect in the testis.

### 3.2 NT‐3 increases the number of Leydig cells in vivo

**Figure 2:**
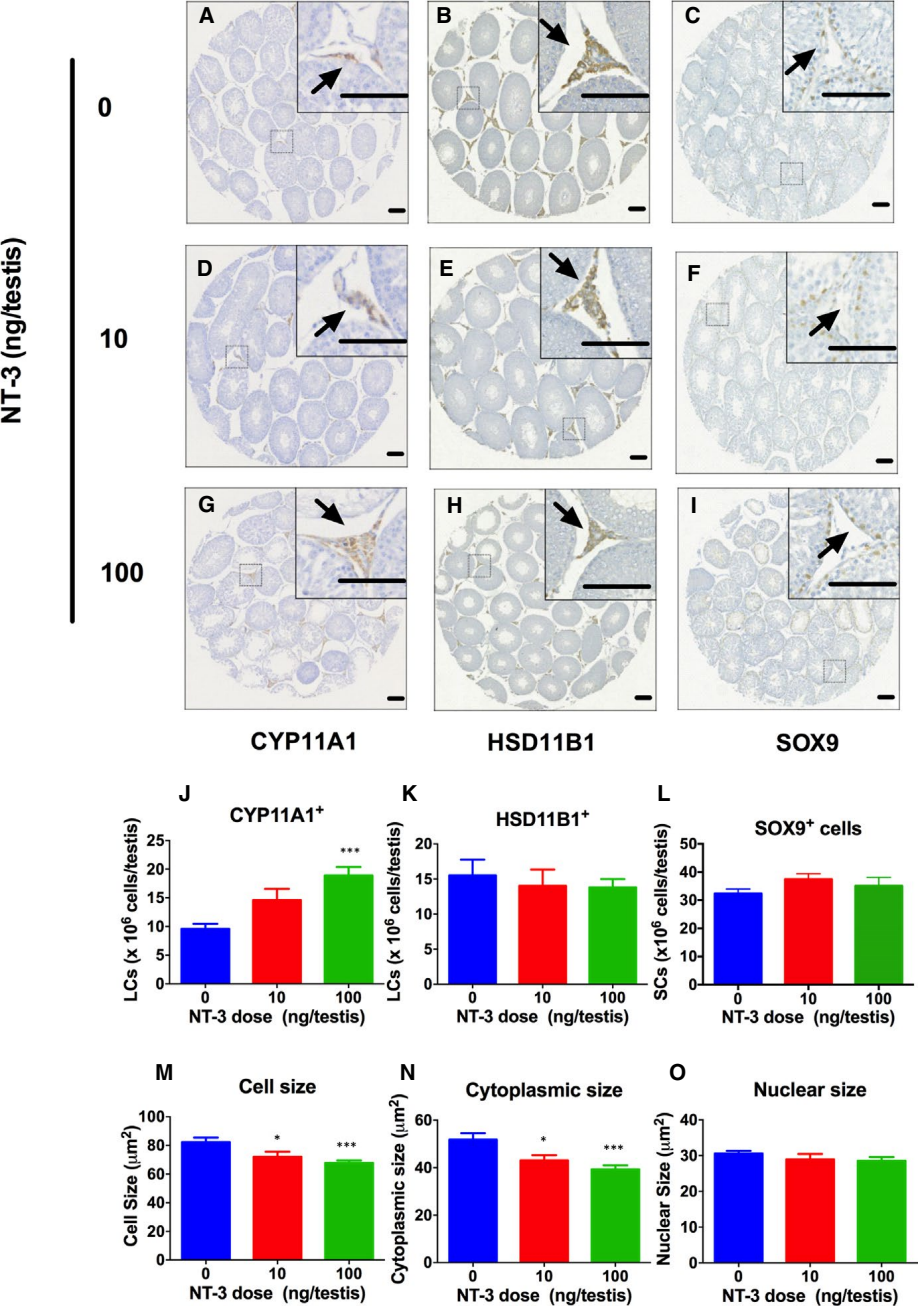
The number of Leydig cells (LC) and Sertoli cells (SC) and Leydig cell metrics in the testes after NT‐3 treatment in vivo. CYP11A1 (Group A, D, G), HSD11B1 (Group B, E, H) and SOX9 (Group C, F, I) testes of rats treated with 0, 10 and 100 ng/testis NT‐3 on the 28th day after EDS. Panels A, B and C: controls; Groups D, E and F: 10 ng/testis NT‐3; Groups G, H and I: 100 ng/testis NT‐3; Panel J, K and L: quantitative data for CYP11A1^+^, HSD11B1^+^ and SOX9^+^ cells; Panel M, N, O: data for LC cell size, cytoplasmic size and nuclear size. The black arrow indicates CYP11A1, HSD11B1 and SOX9 positive cells. Bar = 50 microns. Mean ± SEM, n = 8, compared with control, *P < .05 and ***P < .001

CYP11A1‐positive and HSD11B1‐positive Leydig cells in EDS‐administered testis were counted. CYP11A1 reflects the all Leydig cells and HSD11B1 reflects Leydig cells at the ILC and ALC stage.^6^ It was found that at doses of 10 and 100 ng/testis, NT‐3 dose‐dependently increased the number of CYP11A1‐positive Leydig cells, and the significance was recorded in the 100 ng/testis group (Figure 2A‐L). However, the number of HSD11B1‐positive Leydig cells was not affected by NT‐3. This indicates that NT‐3 stimulates the proliferation of SLC and PLCs. SOX9 is a specific transcription factor exclusively expressed by Sertoli cells.^19^ SOX9 was used as the biomarker to label Sertoli cells. NT‐3 did not affect SOX9 positive Sertoli cell number (Figure 2C, F, I, L).

### 3.3 NT‐3 affects the expression of Leydig cell‐specific genes in vivo

**Figure 3:**
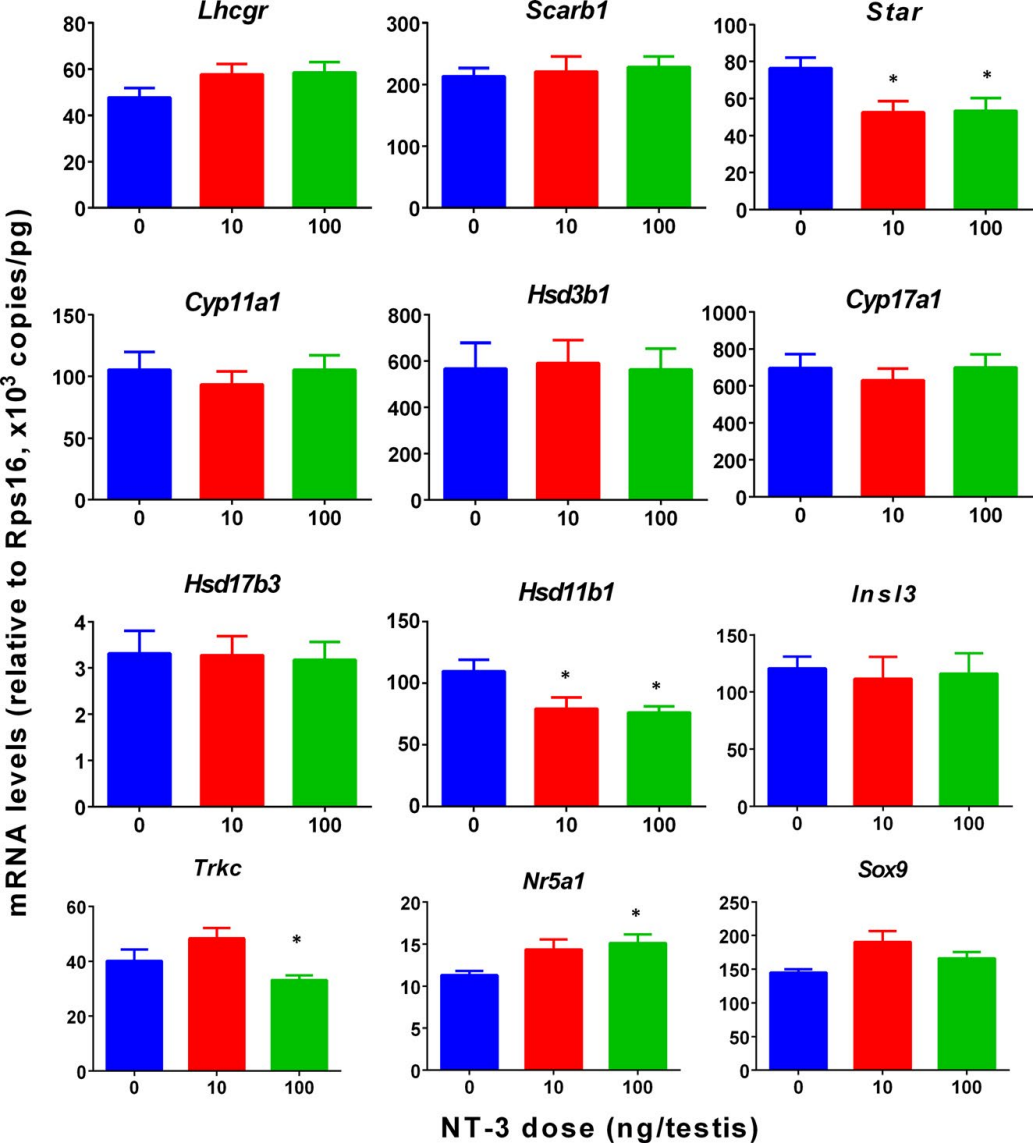
qPCR measurement of mRNA levels in the testes after in vivo NT‐3 treatment. Leydig cell genes: *Lhcgr*, *Scarb1*, *Star*, *Cyp11a1*, *Hsd3b1*, *Cyp17a1*, *Hsd17b3*, *Hsd11b1*, *Insl3*, *Trkc* and *Nr5a1*. Sertoli cell gene: *Sox9*. Mean ± SEM, n = 8. Asterisk (*) designates significant difference from the control (NT‐3, 0 ng/testis) at *P* < .05

The mRNA levels of ALC‐specific genes were measured by qPCR. The results showed that NT‐3 significantly reduced *Star* and *Hsd11b1* mRNA levels at 10 and 100 ng/testis and down‐regulated *Insl3* and *Trkc* expression at a dose of 100 ng/testis compared to the control group (Figure 3). Since Leydig cell number is increased, we re‐analysed the expression of all genes after adjustment to CYP11A1‐positive Leydig cells (*Lhcgr, Scarb1, Star, Cyp11a1, Hsd3b1, Cyp17a1, Hsd17b3, Hsd11b1, Insl3, Trkc* and *Nr5a1*) and SOX9‐positive Sertoli cells (*Sox9*). After adjustment, NT‐3 down‐regulated the expression of all these genes (*Lhcgr, Scarb1, Star, Cyp11a1, Hsd3b1, Cyp17a1, Hsd17b3, Hsd11b1, Insl3, Trkc* and *Nr5a1*) without affecting *Sox9* (Figure S1). This indicates that NT‐3 reduces the level of T down‐regulating the expression of Leydig cell genes. This also indicates that the down‐regulation of *Hsd11b1* is related to the delay of differentiation of SLCs.

### 3.4 NT‐3 affects Leydig cell‐specific protein levels in vivo

**Figure 4:**
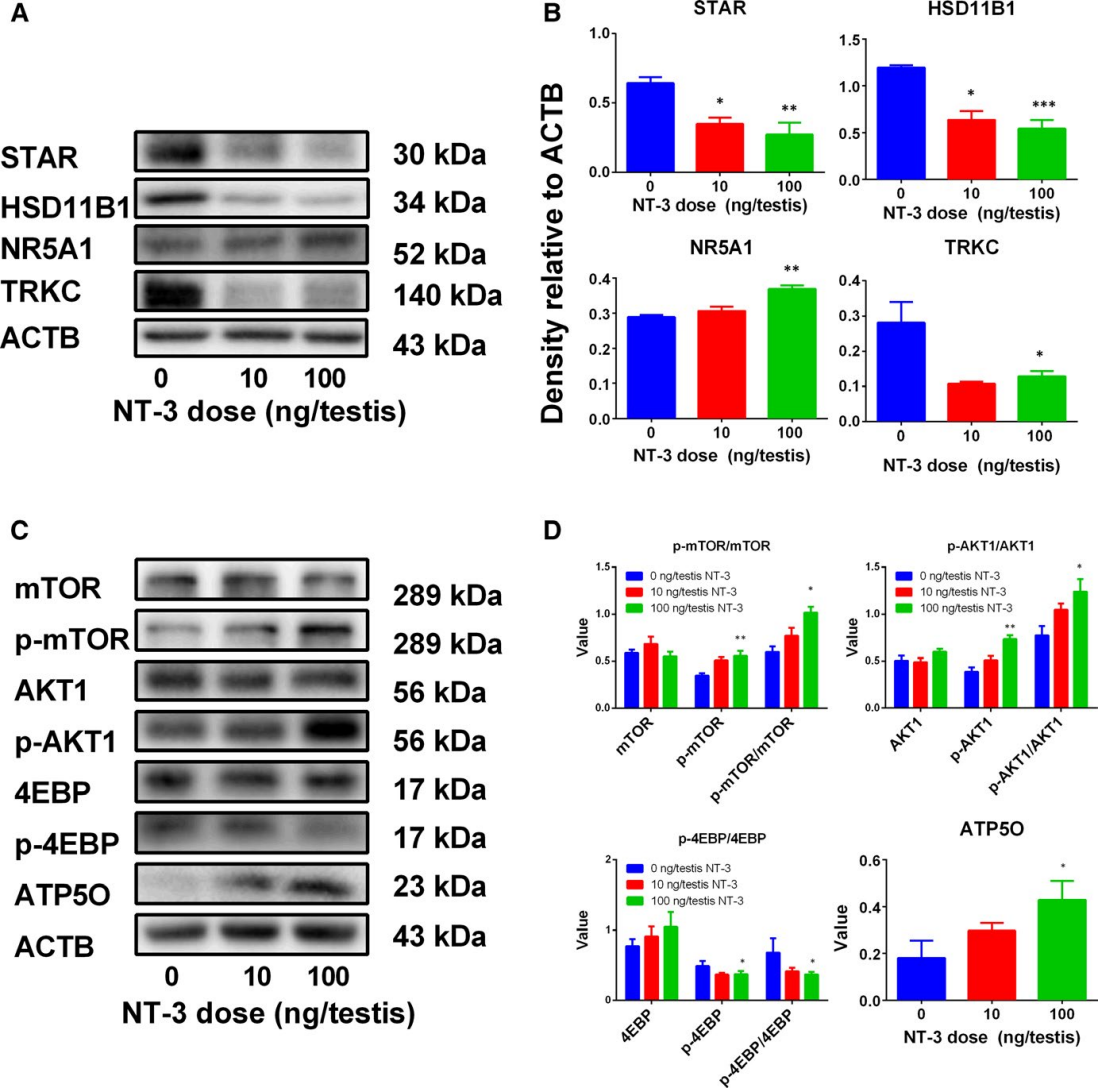
Leydig protein levels from the testes treated with NT‐3 in vivo. Panel A, Western blot image for Leydig cell steroidogenesis‐related proteins and Panel B, their protein quantification; Panel C, Western blot image for signalling pathway proteins and Panel D, their protein quantification. Value represents density relative ACTB of single protein or ratio of phosphorylated protein to the total protein. Mean ± SEM, n = 3‐5. Asterisk (*, **, ***) designate significant difference with the control at *P* < .05, 0.01 and 0.001 respectively

The protein levels of ALCs were detected by Western blotting and it was demonstrated that the protein level change was according to their respective mRNA level (Figure 4). We further used semiquantitative immunohistochemical staining of HSD11B1 and SOX9 (Figure S2). NT‐3 lowered HSD11B1 density without affecting SOX9 density at a dose of 100 ng/testis, further confirming that the expression of HSD11B1 in the individual Leydig cell is down‐regulated.

### 3.5 NT‐3 regulates AKT1 and mTOR pathways in vivo

Proteins in several signalling pathways were detected by Western blot (Figure 4) and it was found that after 100 ng/testis NT‐3, pAKT1/AKT1 and pmTOR/mTOR ratios were increased and then the downstream p4EBP/4EBP ratio was decreased. ATP5O, a mitochondrial transcription‐related gene, was analysed and it was found that its protein level was increased (Figure 4). It is speculated that NT‐3 enhances mitochondrial function and promotes SLC proliferation and blocks the differentiation.

### 3.6 NT‐3 stimulates SLC proliferation in vitro

**Figure 5:**
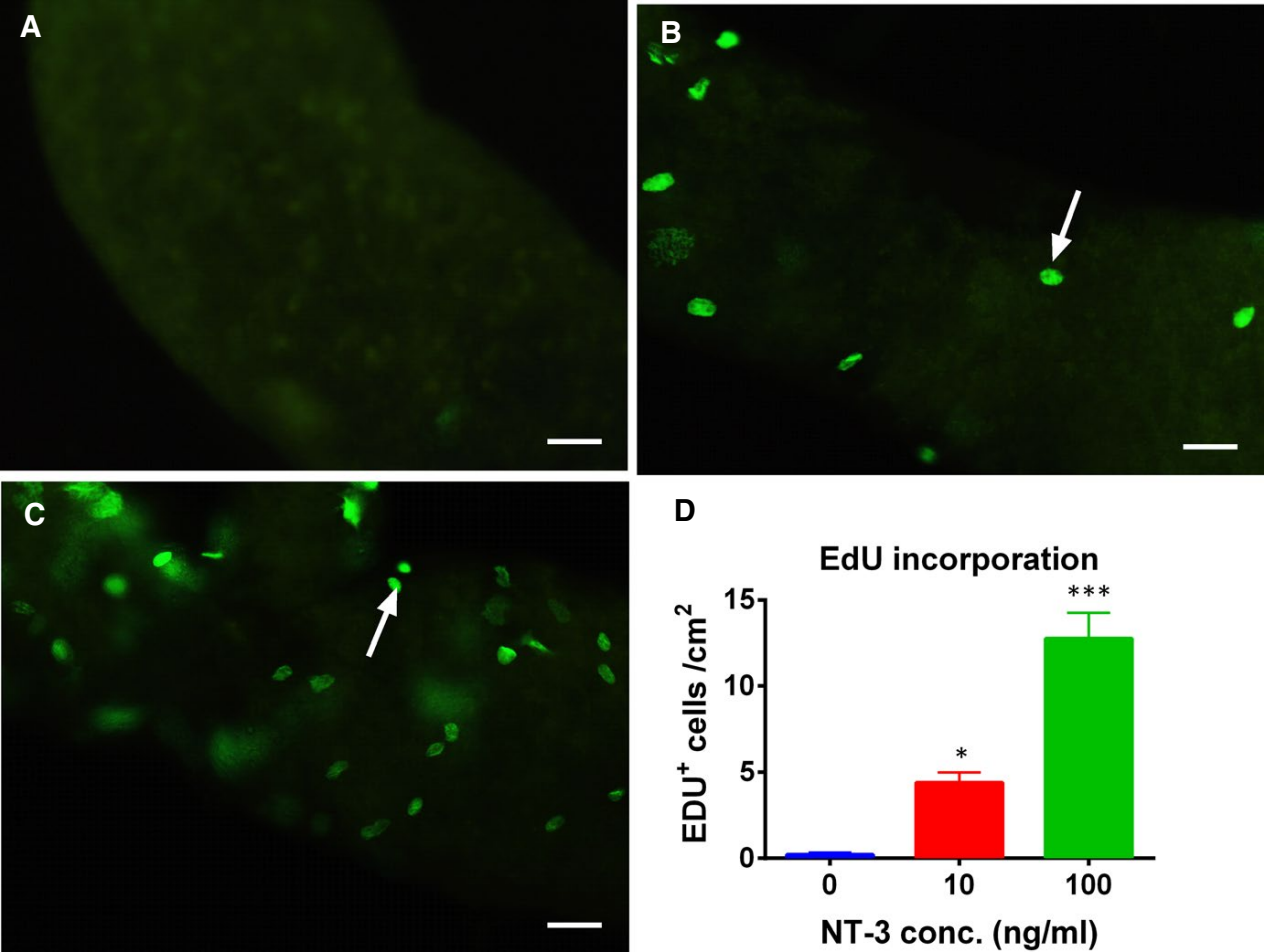
EdU incorporation into SLCs on the surface of seminiferous tubules after NT‐3 treatment in vitro. EdU images (Group A, B, C) for 0, 1 and 10 ng/mL NT‐3 treatment for 3 d. Green colour shows the incorporation of EdU into the nucleus of a proliferating SLC (black arrow). Bar = 20 microns. D: quantitative data for EdU incorporation. Mean ± SEM, n = 6, compared with control, *P < .05 and ***P < .001

In vitro ST culture system was used to investigate the effect of NT‐3 on the proliferation of SLCs.^17^ After 3 days of NT‐3 (1 and 10 ng/mL) treatment, NT‐3 significantly increased the incorporation of EdU into SLCs on the surface of STs at 1 and 10 ng/mL when compared to the control (Figure 5). This indicates that NT‐3 potently stimulate SLC proliferation.

### 3.7 NT‐3 blocks SLC differentiation in vitro

**Figure 6:**
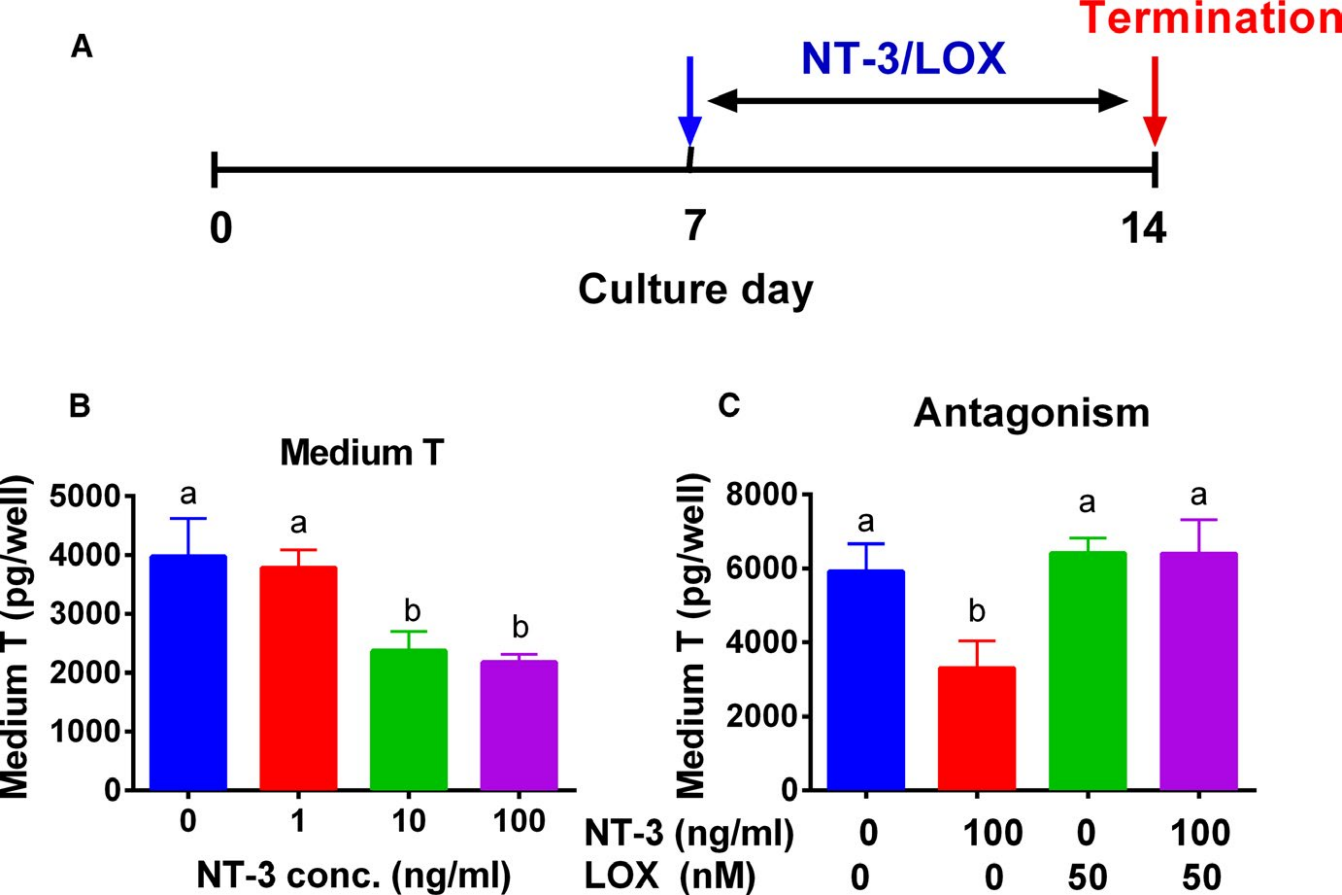
NT‐3 experimental protocol and medium testosterone (T) in vitro NT‐3 treatment. A, Experimental protocol; B, Medium T after different concentrations of NT‐3 treatment; C. Medium T after 100 ng/mL NT‐3 alone plus its antagonist LOXO‐195 (LOX, 50 nmol/L). Mean ± SEM, n = 6. Identical letters indicate that there is no significant difference between two groups at *P* < .05

**Figure 7:**
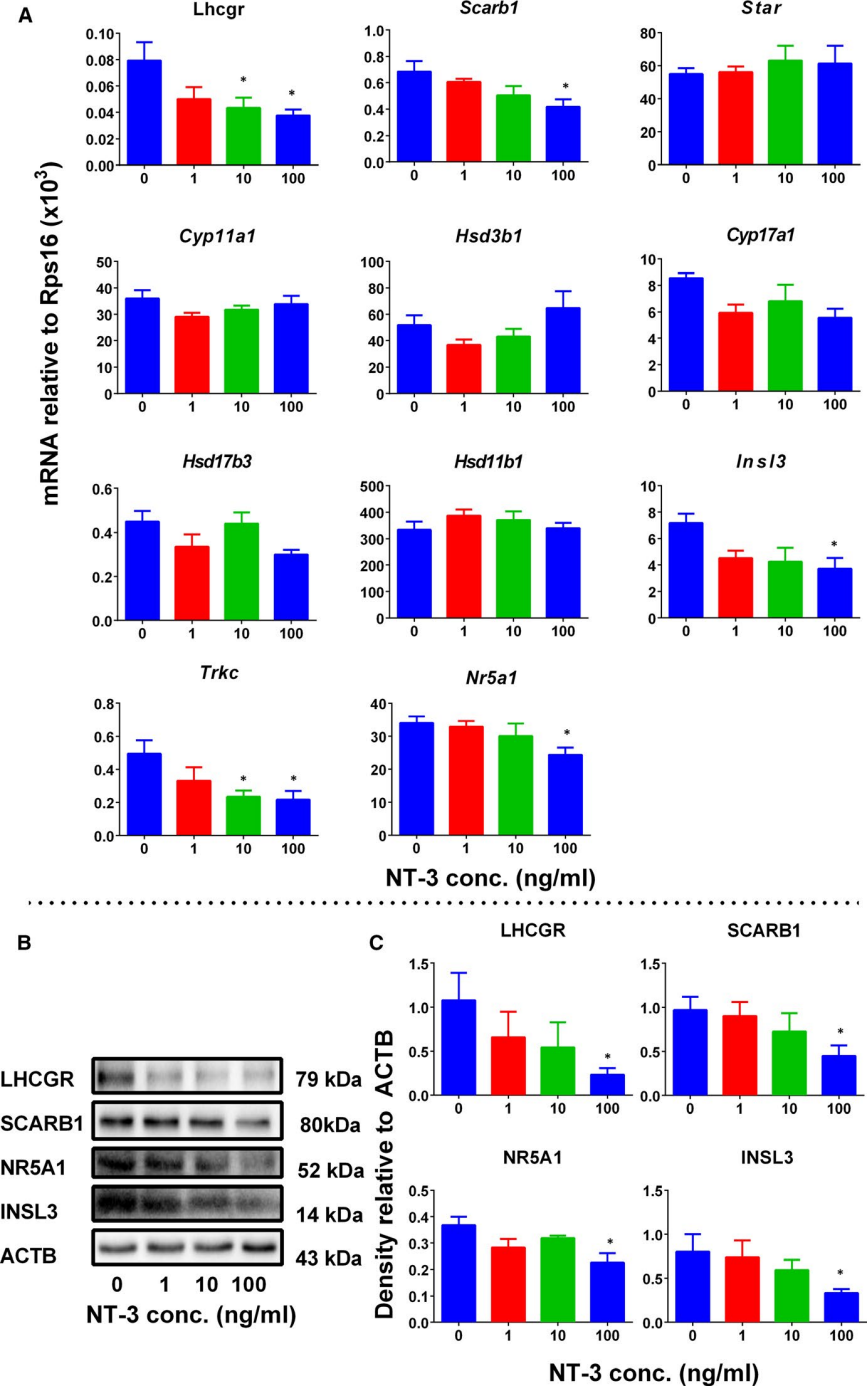
qPCR measurement of mRNA levels and Western blot of their proteins in the testes after in vitro NT‐3 treatment. Panel A, Leydig cell genes: *Lhcgr*, *Scarb1*, *Star*, *Cyp11a1*, *Hsd3b1*, *Cyp17a1*, *Hsd17b3*, *Hsd11b1*, *Insl3*, *Trkc* and *Nr5a1*. Mean ± SEM, n = 6; Panel B and C. Image and quantitative data of proteins: LHCGR, STAR, NR5A1 and INSL3, respectively. Mean ± SEM, n = 3; Asterisk (*) designates significant difference with the control at *P* < .05

In vitro ST culture system was used to investigate the effect of NT‐3 on the differentiation of SLCs.^17^ STs were treated with NT‐3 (1, 10 and 100 ng/mL) alone or together with TRKC antagonist, Celitinib (LOXO‐195, LOX), in LDM for 14 days in vitro (Figure 6A). Indeed, SLCs can be converted into Leydig cells, producing T (Figure 6B). NT‐3 dose‐dependently lowered medium T levels with significance at 10 and 100 ng/mL (Figure 6B). LOX alone did not affect medium T levels while it reversed NT‐3 (100 ng/mL) mediated T suppression (Figure 6C). This suggests that NT‐3 inhibits T production through TRKC receptor. We measured the expression of Leydig cell genes and proteins, and we found that NT‐3 significantly down‐regulated the expression of *Lhcgr, Scarb1, Star, Insl3, Trkc* and *Nr5a1* at 10 and/or 100 ng/mL (Figure 7A). The proteins of LHCGR, SCARB1, TRKC, INSL3 were also lowered by NT‐3 (Figure 7B and C). These further confirm that NT‐3 blocks SLC differentiation into the Leydig cell lineage.

## 4 DISCUSSION

NT‐3 is a member of the NGF family. Previous studies have shown that NT‐3 promoted the growth, proliferation and differentiation of neural stem cells.^20^, ^21^ In addition, NT‐3 is secreted during the embryonic period, and NT‐3 is involved in regulating the formation of seminiferous cords, the differentiation of germ cells, and the determination of male gender.^16^ Our group has also shown that NGF induced SLCs to proliferate and differentiate during ALC regeneration.^11^ Here, we show that NT‐3 stimulates the proliferation of SLCs similar to NGF. NGF has biphasic effects on the differentiation of Leydig cells depending on the Leydig cell stage.^11^ During the stage of SLCs and PLCs, NGF stimulated androgen production by up‐regulating the expression of Leydig cell steroidogenesis‐related genes, while at the ILC stage NGF blocked the differentiation of Leydig cells.^11^ However, unlike NGF, NT‐3 blocks the differentiation of SLCs into the Leydig cell lineage (Figures 6 and 7).

Neurotrophin‐3 has a recognized effect on peripheral nerves and Schwann cells.^22^ In addition, NT‐3 was found to promote the differentiation of mouse myoblasts.^23^ NT‐3 and the receptor TRKC was found to present in mesenchymal cells in foetal rat and human testes.^24^ This indicates that NT‐3 can act on the TRKC receptor and affect the development of rat and human testes.

Ethane dimethyl sulphone can completely kill ALCs in the adult testis of rats and initiate a regeneration of Leydig cells from SLCs.^25^ This EDS model shows two phases: the initial proliferation of SLCs and PLCs and then differentiation into ALCs.^26^ Herein, the development of Leydig cells from SLCs after 28 days of EDS treatment was studied for NT‐3. We found that NT‐3 significantly increased the number of CYP11A1‐positive Leydig cells in vivo (Figure 2). CYP11A1 is expressed in all cells in the Leydig cells.^27^ This suggests that the total number of Leydig cells is increased after NT‐3 treatment. However, the HSD11B1‐positive Leydig cell number was not increased by NT‐3. HSD11B1 is only expressed in ILCs and ALCs,^6^, ^28^ suggesting that some of Leydig cells are not HSD11B1‐positive and they are in the PLC stage. We also confirmed the proliferation of SLCs by the incorporation of EdU assay in vitro (Figure 5). These results indicate that NT‐3 promotes the proliferation of SLCs to increase Leydig cell population.

Our experiments found that NT‐3 can effectively inhibit serum T levels in vivo (Figure 1) without affecting serum LH and FSH levels. We used intratesticular injection of NT‐3 to avoid the systemic effects of NT‐3 on the hypothalamic‐pituitary‐gonadal axis. Although we did not observe a significant change of serum LH levels, it trended higher especially in the highest NT‐3 group due to negative feedback. In this EDS model, due to the elimination of Leydig cells, T declined to an almost undetectable level 2 days after EDS, while LH rose to the peak level 14 days after EDS.^29^ When Leydig cells began to regenerate 14 days after EDS, T started to rise and reached the pretreatment level about 21‐28 days after EDS, and LH started to rise 2 days after EDS and reached the peak level 28 days after EDS but remained at a high level until post‐EDS day 42.^29^ Due to the decline of T, spermatogenesis was hindered, FSH level rose to the peak through negative feedback, and maintained the peak until T and spermatogenesis recovered, then serum FSH returned to normal level 49 days after EDS.^29^ In this study, we found that serum FSH level was 6 ng/mL in the control, which was higher than the normal serum level (about 2‐3 ng/mL).^29^ Although serum T rose on post‐EDS day 28 in the control but serum FSH levels were still higher, indicating that spermatogenesis is low in both control and NT‐3 treated groups.

Quantitative analysis of mRNAs showed that at a dose of 100 ng/testis, NT‐3 significantly reduced the levels of *Star* and *Hsd11b1* mRNAs. Since CYP11A1‐positive Leydig cells were increased, we re‐analysed mRNAs after adjustment to CYP11A1‐positive Leydig cell number and found that the expression of all Leydig cell genes was down‐regulated (Figure S1). STAR is the rate‐limiting protein for cholesterol transportation in T synthesis pathway.^30^ It can transport cholesterol to the mitochondrial inner membrane, thereby catalysing the synthesis of pregnenolone by CYP11A1 enzyme.^31^ HSD11B1 is one of the biomarkers of Leydig cells at the advanced stages such as ILC and ALC stages.^6^, ^28^ The down‐regulation of *Hsd11b1* indicates that the differentiation of Leydig cells is delayed.

Seminiferous tubules were cultured together with NT‐3 alone or together with its antagonist LOX in vitro. NT‐3 inhibited the differentiation of SLCs into the Leydig cell lineage in vitro, which is manifested by the decrease of T content in the medium and the decrease of expression of Leydig cell genes (Figure 7). Our in vitro studies also clearly showed that after using TRKC antagonist (LOXO‐195), NT‐3 exerted its inhibitory effect on SLC differentiation through the NT‐3/TRKC signalling pathway.

Among the various functions of the phosphatidylinositol 3‐kinase (PI3K)/ protein kinase B (AKT1)/ mammalian target of the cascade of rapamycin (mTOR), cell proliferation is important. In this cascade, PI3K and AKT1 is a key upstream molecule that links the connection of growth factor receptors such as insulin receptor or insulin‐like growth factor receptor after binding of their growth factors to the phosphorylation and activation status of mTOR.^32^ The PI3K pathway can be activated in response to different growth factors (such as insulin‐like growth factor 1, IGF1), cytokines (interleukin 6) and hormones, resulting in the production of phosphatidylinositol 3‐5 triphosphate (PIP3), which absorbs inactive AKT1 from the cytoplasm to the plasma membrane, where AKT1 passes through double phosphorylation at Thr308 and Ser473 and then activated Akt phosphorylates mTOR and its downstream signalling molecules, leading to protein translation.^32^ Therefore, we also explored the possible impact of NT‐3 on AKT1/mTOR signalling pathway. AKT1 is particularly important for the growth and proliferation of cells.^33^, ^34^ AKT1 is an important mediator for cell proliferation linked with growth factor like IGF1.^35^ Akt1 gene knockout has been found to affect the development of mouse testes.^36^ IGF1 has been found to be an important growth factor that affect Leydig cell development postnatally.^37^ IGF1 can stimulate Leydig cell proliferation and inhibit Leydig cell apoptosis by increasing the phosphorylation of AKT1.^38^ IGF1 gene knockout animal models have also been found to completely block the proliferation of Leydig cells^39^ and affect the expression levels of certain Leydig cell‐specific genes.^37^, ^40^ Indeed, we found that NT‐3 significantly increased the phosphorylation of AKT1 (Figure 4), thus possibly stimulating SLC proliferation.

AKT1 may acts via the downstream mTOR pathway.^41^ Previous studies have shown that NT‐3 can regulate cell proliferation through the mTOR pathway.^42^ mTORC1 prevents 4EBP, a transcription factor, from binding to eIF4E and promotes the translation of mRNA encoding mitochondrial‐related proteins, thereby promoting the production of mitochondrial energy.^43^ Here, we found that NT‐3 down‐regulated the 4EBP phosphorylation, thus increasing mitochondrial ATP5O activity (Figure 4). ATP5O is an important mitochondrial protein for mitochondrial activity.^44^ It can be seen that NT‐3 may play a role in the AKT1/ mTOR/ 4EBP pathway, increasing mitochondrial activity and triggering cell proliferation. TRKC overexpression will affect neural stem cell migration. Quantitative analysis of *Trkc* mRNA in vivo (Figure 3) and in vitro (Figure 7) revealed that *Trkc* expression was significantly reduced at a dose of 100 ng/testis NT‐3 in vivo or 10‐100 ng/mL NT‐3 in vitro. This means that NT‐3 may lead to a decrease in TRKC receptors, thereby affecting the normal development of SLCs.

In summary, we demonstrate that NT‐3 binds to TRKC to stimulate the proliferation of SLCs, and block the differentiation of SLCs into the Leydig cell lineage. After binding to TRKC, NT‐3 may activate the AKT1/ mTOR

pathway.

## CONFLICT OF INTEREST

The authors declare that they have no conflict of interest to declare.

## AUTHOR CONTRIBUTION

**Yige Yu:** Conceptualization (lead); Data curation (lead); Formal analysis (lead); Investigation (equal); Methodology (equal); Project administration (lead); Resources (equal); Software (lead); Supervision (lead); Validation (lead); Visualization (lead); Writing‐original draft (lead); Writing‐review & editing (equal). **Zengqiang Li:** Conceptualization (equal); Data curation (equal); Formal analysis (equal); Investigation (equal); Methodology (equal); Resources (equal); Software (equal); Supervision (equal); Validation (equal). **Feifei Ma:** Conceptualization (equal); Data curation (equal); Formal analysis (equal); Investigation (equal); Methodology (equal); Resources (equal); Software (equal); Supervision (equal); Validation (equal); Visualization (equal). **Quanxu Chen:** Formal analysis (equal); Investigation (equal); Methodology (equal); Supervision (equal); Validation (equal). **liben lin:** Data curation (equal); Investigation (equal); Methodology (equal); Resources (equal); Software (equal). **Qiang Xu:** Data curation (equal); Investigation (equal); Resources (equal); Software (equal); Supervision (equal); Validation (equal). **Yang Li:** Conceptualization (equal); Data curation (equal); Formal analysis (equal); Investigation (equal); Resources (equal); Software (equal). **Peipei Pan:** Conceptualization (equal); Formal analysis (equal); Investigation (equal); Software (equal); Supervision (equal). **Xiu Xin:** Resources (equal); Software (equal); Supervision (equal); Validation (equal). **Tongliang Huang:** Conceptualization (equal); Data curation (equal); Investigation (equal); Supervision (equal); Validation (equal). **Yiyan Wang:** Formal analysis (equal); Methodology; Resources (equal); Software (equal); Supervision; Validation; Visualization (equal). **Qianjin Fei:** Conceptualization (equal); Data curation (equal); Resources; Software (equal); Supervision; Validation. **Renshan Ge:** Conceptualization (equal); Data curation (equal); Formal analysis (equal); Funding acquisition (lead); Investigation (equal); Methodology (equal); Project administration (equal); Resources (equal); Software (equal); Supervision (equal); Validation (equal); Visualization (equal); Writing‐original draft (equal); Writing‐review & editing (equal).

## Supporting information

FigS1

FigS2

SupInfoS1

SupInfoS2

SupInfoS3

SupInfoS4

SupInfoS5

## Data Availability

The data that supports the findings of this study are available in the supplementary material of this article
