# Supplementary figures and images for: Neurotrophin‐3 stimulates stem Leydig cell proliferation during regeneration in rats
Source: J Cell Mol Med. 2020 Oct 22;24(23):13679–89. doi: 10.1111/jcmm.15886 (PMC7753877; doi:10.1111/jcmm.15886)

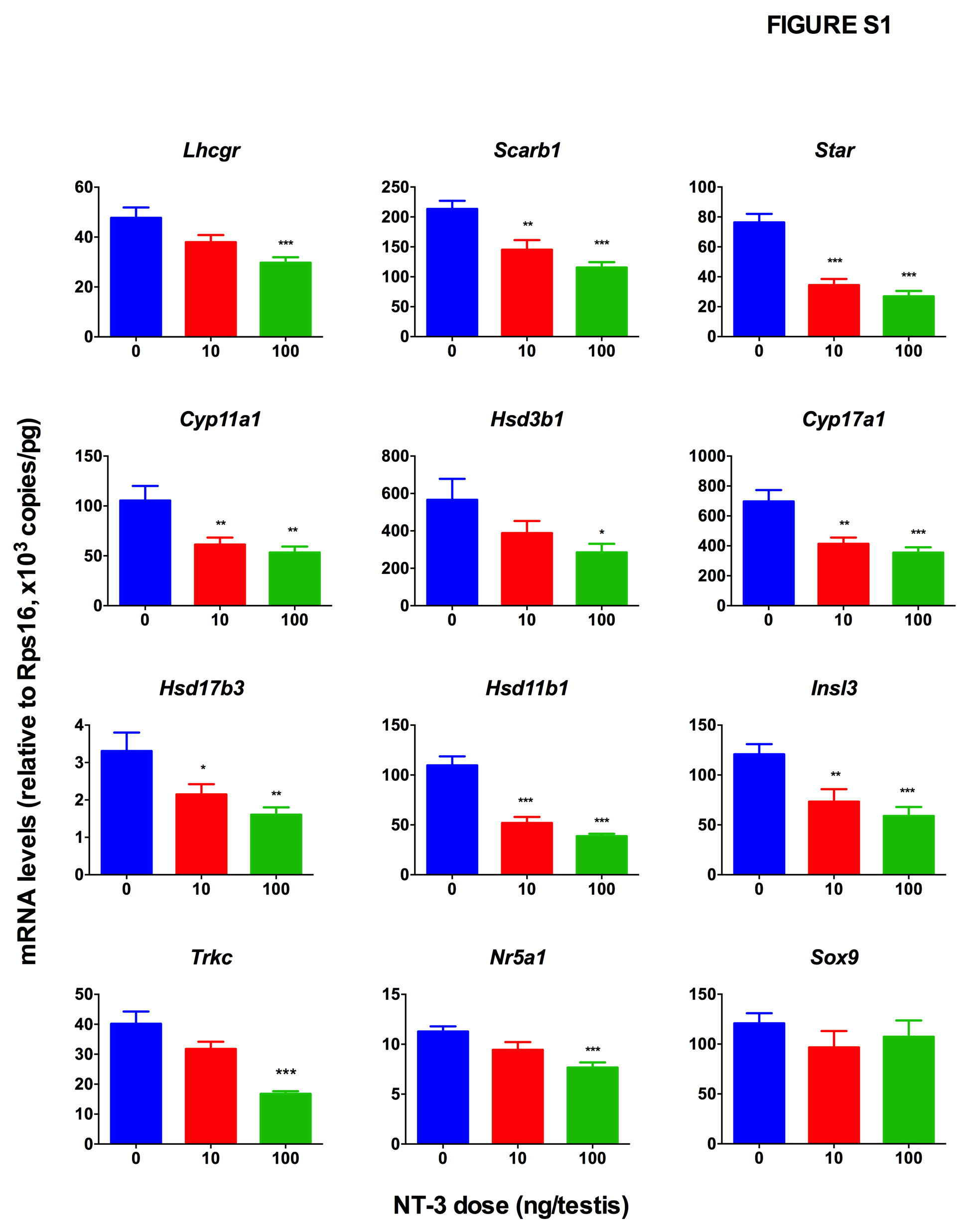

Supplement: Supplementary file 1 — FigS1 [file JCMM-24-13679-s001.tiff]

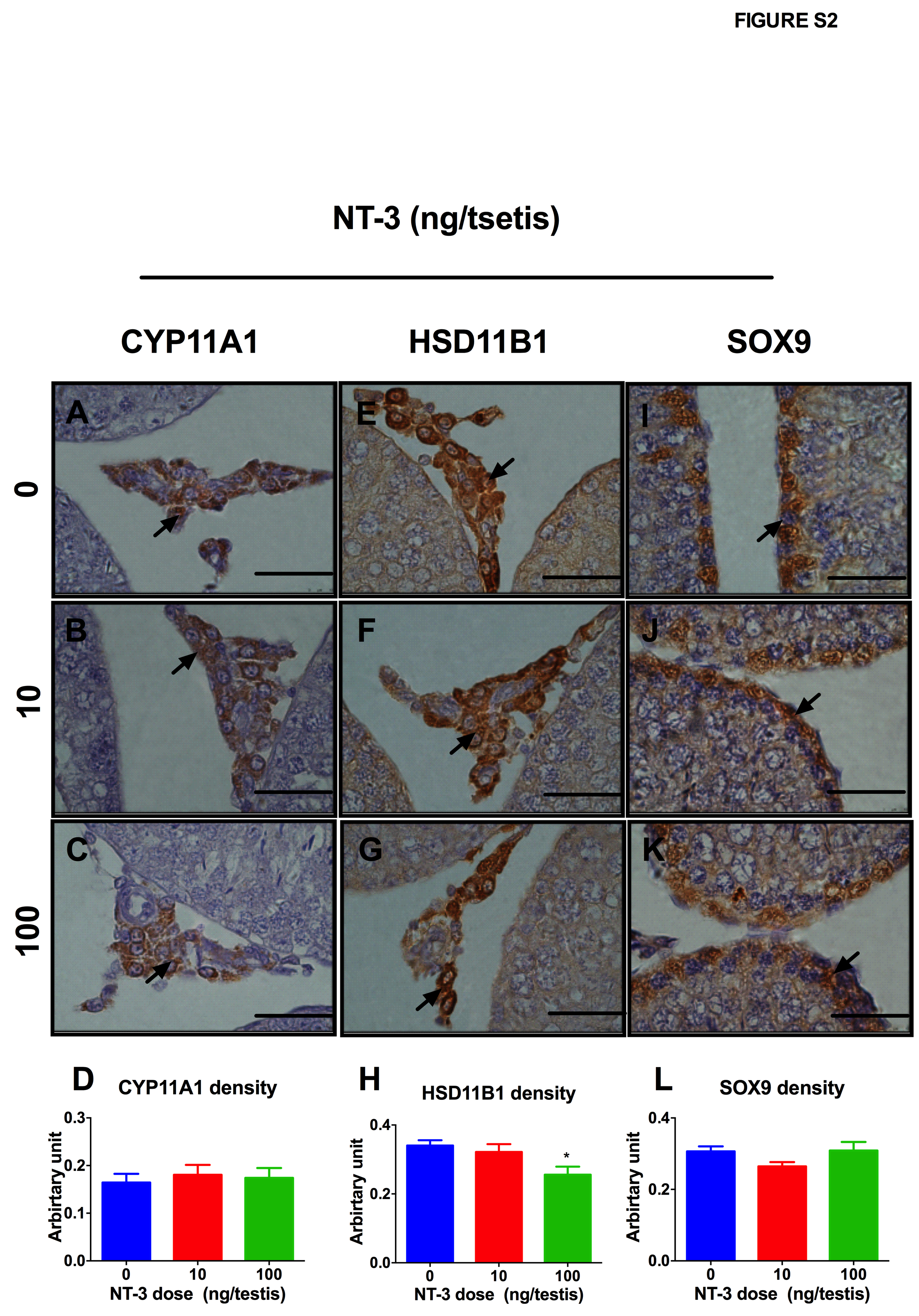

Supplement: Supplementary file 2 — FigS2 [file JCMM-24-13679-s002.tiff]
