# Supplementary material for: Neurotrophin‐3 stimulates stem Leydig cell proliferation during regeneration in rats
Source: J Cell Mol Med. 2020 Oct 22;24(23):13679–89. doi: 10.1111/jcmm.15886 (PMC7753877; doi:10.1111/jcmm.15886)
Supplement: Supplementary file 3 — SupInfoS1 [file JCMM-24-13679-s003.docx]

**2** | **Materials and Method**

**2.1** | **Chemicals and kits**

Details of materials and methods are contained in supplementary material S1. Chemicals, test kits, equipment and software are included in supplementary material S2. The primers used for gene expression are included in supplementary material S3. Supplementary material S4 contains antibodies for immunohistochemical staining and Western blotting. Celitinib (LOXO-195, LOX) is an NT-3 inhibitor.

**2.2**| **Animal study for EDS-treated SLC regeneration**

Twenty-four male Sprague Dawley rats were transported to Wenzhou Medical University and adapted for a week under the new environment. To kill adult Leydig cell (ALC) in the testes, each rat was given ethane dimethane sulfonate (EDS, 75 mg/kg body weight) intraperitoneally. The solvent of EDS is dimethyl sulfoxide: water (1: 3, v / v). Male rats were randomly divided into three groups, 8 in each group. From the 14th to the 28th day after EDS administration, we injected 0 (normal saline), 10 or 100 ng/testis NT-3 into each testis. The injection volume within the testis was 20 μl, and preliminary data showed that the volume did not affect the testis function. In order to rule out the systemic effects caused by hypothalamus-pituitary-testis axis we chose the intratesticular injection protocol. On the 14th day after NT-3 treatment, rats were euthanized with carbon dioxide, and blood samples were collected for serum analysis of hormones. We measured the levels of T, LH and follicle-stimulating hormone (FSH) in these serum samples. We collected total RNAs from the testis for real-time quantitative PCR (qPCR) and Western blot analysis. The contralateral testis was immersed in a bottle containing Bouin's solution and used for immunohistochemical staining. The animal experiment protocol was approved by the Animal Protection and Use Committee of Wenzhou Medical University.

**2.3** | **Determination of serum and medium T**

Both serum and medium T concentration was detected by Immulite2000 Total Testosterone kit as previously mentioned.^1^ Normal male rat serum (2 ng/ml) was used as internal quality control. The minimum determine concentration of T is 0.2 ng / ml.

**2.4** | **ELISA for serum LH and FSH Levels**

LH and FSH levels were assayed using ELISA kits as described previously.^1^ Serum sample and assay solution were mixed and incubated for 2 hours at room temperature. Peroxidase-conjugated IgG anti-LH or anti-FSH liquid was then mixed and incubated for 2 hours at room temperature. The substrate buffer was followed and the plate was stored in the dark place for 30 minutes. The reaction stop solution was added to end the enzyme reaction. The parameters of microplate reader were setup as 550 nm with correction wavelength at 450 nm.

**2.5** | **qPCR**

A testicle each rat was picked and put in Trizol solution, total RNAs were extracted as previously mentioned.^1^ After purifying the RNA, NanoDrop 2000 was used to read the RNA concentration. The cDNAs of these RNA samples were reverse transcribed according to the method previously described.^1^ The SYBR Green qPCR kit (Roche, Basel, Switzerland) was used to measure the following Leydig cell mRNAs: *Lhcgr*, *Scarb1*, *Star*, *Cyp11a1*, *Hsd3b1*, *Cyp17a1*, *Hsd17b3*, *Insl3*, *Hsd11b1, Trkc*, *Nr5a1*, and *Sox9*. 7.5 μl SYBR Green Mix, 0.75 μl forward primer / 0.75 μl reverse primer, 0.02 μg diluted cDNA and 4 μl RNase-free water were mixed together. The qPCR program includes 95 ° C for 5 min, 40 cycles of 95 °C for 10 s and 60 °C for 30 s. The melting curve analysis and gel electrophoresis were selected to identify PCR specificity. A standard curve using Ct values was generated to calculate the concentration of target mRNA. The target mRNA level was adjusted to *Rps16*. *Rps16* is used for internal control. Supplementary table S1 includes primer information.

**2.6** | **Western blot analysis**

The Western blot technique was selected as previously described.^1^ Briefly, a testicle or ST was cut, then homogenized in a glass homogenizer. A BCA protein assay kit (Japan, Takara) was selected to measure protein concentration. 30 μg of protein from each sample was loaded into the gel and separated using denaturing electrophoresis. After electrophoresis, the protein is transferred to the nitrocellulose membrane. 5% skim milk is used to block non-specific binding. The following primary antibodies, LHCGR, SCARB1, STAR, HSD11B1, NR5A1, phosphorylated AKT1 (pAKT1), AKT1, phosphorylated mTOR (pmTOR), mTOR, phosphorylated 4EBP-1 (p4EBP), 4EBP, and ATP5O, were incubated, respectively. After washing with several washes, the membrane was incubated with HRP-conjugated anti-rabbit or anti-mouse (1: 2,000, Bioword, USA) for 2 hours. Chemiluminescent substrate was added, and photos of the band were taken. ACTB is an internal protein control. Band intensity is read. The data of the target protein was normalized to ACTB.

**2.7** | **Preparation of testis tissue array for immunohistochemical staining and stereological counting of cells**

A tissue-array was prepared as described previously.^1^ One testicle per rat (8 rats per group) was picked, cut into 8 discs, and two discs were randomly selected. The two discs were further cut into four pieces, and one of the eight pieces was randomly selected. The testis-block was embedded in the tissue-array container with paraffin. The tissue-array block was cut into 5μm thick sections. From these sections, 10 slides were randomly selected for immunohistochemical staining, and 10 other slides were selected for immunofluorescence staining.

**2.8** | **Immunohistochemistry and immunofluorescence staining of testis**

According to the previously reported method,^1^ Immunohistochemical staining kit was used. Tissue slide was performed for antigen recovery in 10 mM (pH 6.0) citrate buffer in a microwave oven. The slide was immerged in 0.5% H_2_O_2_ in methanol for 30 minutes to remove endogenous peroxidase activity. Three antibodies were used, polyclonal antibodies to CYP11A1 (a biomarker for the Leydig cell lineage) or HSD11B1 (a biomarker of Leydig cells at the ILC and ALC stages), as well as SOX9 for Sertoli cells. The antibody was diluted 1: 200 (v/v). Diaminobenzidine solution was added to show the brown color of the target protein. The counterstain is Mayer hematoxylin. Non-immunized rabbit IgG was used as a negative control. Immunofluorescence staining on PCNA and CYP11A1 was performed to identify hyperplastic Leydig cells. The Alexa-conjugated anti-rabbit or anti-mouse IgG secondary antibody (1: 500) was selected. Counterstaining is a DAPI solution. Positive cells were identified using Olympus fluorescence microscope (Olympus Japan). CYP11A1 (green in the cytoplasm) represents Leydig cells, and PCNA (red in the nucleus) represents proliferating cells.

**2.9** | **Calculation of Leydig cell and Sertoli cell number per testis**

In order to count CYP11A1-positive or HSD11B1-positive Leydig cells as well as SOX9-positive Sertoli cells, the fractionator technique was used for the above tissue-array section as described.^1^ Briefly, under the real-time image of a digital camera with a 10× objective lens and a fixed point in the "upper" part, the cells of the total microscopic field of view was calculated.

**2.10** | **ST culture for SLC developmental assay**

To investigate whether NT-3 can affect the development of SLCs, an in vitro culture system of SLCs on the surface of STs was used as previously mentioned.^1^ 90-day-old male Sprague Dawley rats were treated with EDS (75 mg/kg body weight). On day 7 after EDS, the rats were killed by carbon dioxide and Leydig cells were depleted. The testis was placed in MEM-199. STs were separated mechanically. STs were cut into pieces about one inch long and randomly assigned to a 12-well plate. Each well contains an equal amount of STs. The STs were cultured with LDM containing 5 ng/mL LH, 5 mmol/L insulin-transferrin-selenium, and 5 mmol/L lithium chloride in DMEM: F12 medium (1: 1, v/v, PH 7.2) supplemented with 0.1% bovine serum albumin, 15 mmol/L HEPES, 2.2 mg/mL sodium bicarbonate and penicillin/streptomycin (100 U/mL and 100 mg/mL). The plate was placed at 37° C in a humidified atmosphere of 5% CO_2_ for 2 weeks. At the end of 2 weeks, the SLCs differentiate into ALCs, which can secrete T into the culture medium and T can be detected. NT-3 was added to LDM. Medium T concentrations were determined as above.

**2.11** | **Incorporation of EdU into SLCs**

As mentioned earlier,^1^ the incorporation of EdU into SLCs is measured by EdU Alaxa Fluor kit (Life Technologies, USA. In brief, freshly isolated STs were cultured in M199 medium as above and treated with 0–100 ng/ml NT-3 for 7 days. EdU (1: 1,000 dilution) was added to the STs and incubated for another 24 hours. The ST was washed with PBS containing 3% BSA. ST was fixed in 4% paraformaldehyde for 30 minutes. The ST was washed and stained. Images were captured using an Olympus fluorescence microscope (Olympus Japan). Image-Plus software (Media Cybernetics, Rockville, MD) was used to count EdU-positive cells (green in the nucleus).

**2.12** | **Statistical analysis**

The data are presented using the mean ± SEM. P <0.05 was considered statistically significant. One-way ANOVA then Sidak-adjusted Dunnett multiple comparison test or paired student t test (only for Western blotting analysis) in SigmaStat software (Richmond, CA) was used to compare them with controls.

1. **Wang Y, Xie L, Tian E, Li X, Wen Z, Li L, Chen L, Zhong Y, Ge RS.** Oncostatin M inhibits differentiation of rat stem Leydig cells in vivo and in vitro. *Journal of cellular and molecular medicine*. 2019; 23: 426-38.
