# Supplementary material for: Neurotrophin‐3 stimulates stem Leydig cell proliferation during regeneration in rats
Source: J Cell Mol Med. 2020 Oct 22;24(23):13679–89. doi: 10.1111/jcmm.15886 (PMC7753877; doi:10.1111/jcmm.15886)
Supplement: Supplementary file 4 — SupInfoS2 [file JCMM-24-13679-s004.docx]

Supplementary material S2. Chemicals, regents, test kits, equipment, software, and service

| **Name** | **Vendor (City, State)** |
| --- | --- |
| Sprague Dawley rats (age 56 days) | Shanghai Laboratory Animal Center (Shanghai, China) |
| Recombinant neurotrophin-3 | PeproTech (Rocky Hill, NJ) |
| Celitinib | MedChemExpress (Monmouth Junction, NJ) |
| Trizol | Invitrogen (Carlsbad, CA) |
| SYBR Green qPCR Kit | Takara (Otsu, Japan) |
| Click-iT EdU imaging kit | Invitrogen (Carlsbad, CA) |
| Ethane dimethane sulfonate | Pterosaur Biotech (Hangzhou, China) |
| BCA Protein Assay Kit | Takara (Otsu, Japan) |
| Immulite2000 Total Testosterone Kit | Sinopharm (Hangzhou, China) |
| LH ELISA kit | Chemicon (Temecula, CA) |
| FSH ELISA kit | Chemicon (Temecula, CA) |
| NanoDrop 2000 | Thermo Fisher Scientific (Springfield, NJ) |
| RNA-seq | Aksomics Inc (Shanghai, China) |
| Vector ABC Kit | Vector (Burlingame, CA) |
| BX53 microscope | Olympus (Tokyo, Japan) |
| Image-Pro 6 Plus software | Media Cybernetics (Silver Spring, MD) |
| Universal Hood II | Bio-Rad (Hercules, CA) |
| Super-Signal West Pico substrate | Pierce Biotechnology (Radford, IL) |
| Image Lab (Hercules, CA). | Bio-Rad (Hercules, CA) |
| GraphPad Prism | GraphPad (San Diego, CA) |
