# Supplementary material for: Neurotrophin‐3 stimulates stem Leydig cell proliferation during regeneration in rats
Source: J Cell Mol Med. 2020 Oct 22;24(23):13679–89. doi: 10.1111/jcmm.15886 (PMC7753877; doi:10.1111/jcmm.15886)
Supplement: Supplementary file 5 — SupInfoS3 [file JCMM-24-13679-s005.docx]

**Supplementary material S3. Primer information**

| **Primer**  **Symbol** | **Gene name** | **Primer direction** | **Sequences (5’to 3’)** | **PCR**  **(bp)** | **Accession** |
| --- | --- | --- | --- | --- | --- |
| Lhcgr | Luteinizing hormone receptor | Forward | CTGCGCTGTCCTGGCC | 103 | NM_012978 |
|  |  | Reverse | CGACCTCATTAAGTCCCCTGAA |  |  |
| Scarb1 | Scavenger receptor class B, member 1 | Forward | ATGGTACTGCCGGGCAGAT | 117 | NM_031541 |
|  |  | Reverse | CGAACACCCTTGATTCCTGGTA |  |  |
| Star | Steroidogenic acute regulatory protein | Forward | CCCAAATGTCAAGGAAATCA | 187 | NM_031558 |
|  |  | Reverse | AGGCATCTCCCCAAAGTG |  |  |
| Cyp11a1 | Cholesterol side chain cleavage enzyme | Forward | AAGTATCCGTGATGTGGG | 127 | NM_017286 |
|  |  | Reverse | TCATACAGTGTCGCCTTTTCT |  |  |
| Hsd3b1 | 3β-Hydroxysteroid dehydrogenase 1 | Forward | CCCTGCTCTACTGGCTTGC | 189 | NM_001007719 |
|  |  | Reverse | TCTGCTTGGCTTCCTCCC |  |  |
| Cyp17a1 | P450 17α-hydroxylase/ | Forward | TGGCTTTCCTGGTGCACAATC | 90 | NM_012753 |
|  | 17,20-lyase | Reverse | TGAAAGTTGGTGTTCGGCTGAAG |  |  |
| Insl3 | Insulin-like 3 | Forward | GTGGCTGGAGCAACGACA | 102 | NM_053680 |
|  |  | Reverse | TGAAAGTTGGTGTTCGGCTGAAG |  |  |
| Hsd17b3 | 17β-Hydroxysteroid  dehydrogenase 3 | Forward | TGAAAGTTGGTGTTCGGCTGAAG | 202 | NM_054007 |
|  |  | Reverse | CCACAAGCCAATACAAACTAACT |  |  |
| Sox9 | SRY box 9 | Forward  Reverse | TGCTGAACGAGAGCGAGAAG  ATGTGAGTCTGTTCGGTGGC | 160 | NM_080403.1 |
| Nr5a1 | nuclear receptor subfamily 5, group A, member 1 | Forward  Reverse | CAGAGCTGCAAAATCGACAA  CCCGAATCTGTGCTTTCTTC | 186 | NM_001191099.1 |
| Trkc | neurotrophic tyrosine kinase receptor type 3 | Forward  Reverse | CACTTGTAATGGCTCTGGCTCTCC  TTGTCTTCGCTCGTCACATTCACC | 147 | NM_001270565.1 |
| Rps16 | Ribosomal protein  S16 | Forward | AAGTCTTCGGACGCAAGAAA | 148 | [NM_001169146](https://www.ncbi.nlm.nih.gov/entrez/viewer.fcgi?db=nucleotide&id=310703681) |
|  |  | Reverse | TTGCCCAGAAGCAGAACAG |  |  |
