# Supplementary material for: Neurotrophin‐3 stimulates stem Leydig cell proliferation during regeneration in rats
Source: J Cell Mol Med. 2020 Oct 22;24(23):13679–89. doi: 10.1111/jcmm.15886 (PMC7753877; doi:10.1111/jcmm.15886)
Supplement: Supplementary file 6 — SupInfoS4 [file JCMM-24-13679-s006.docx]

**Supplementary material S4. Antibodies**

| **Antibody** | **Species** | **Vendor (City, State, catalogue)** | **Dilution** | |
| --- | --- | --- | --- | --- |
|  |  |  | **WB** | **HS** |
| LHCGR | rabbit | Multi Sciences (Hangzhou, China) | 1:1000 | ND |
| SCARB1 | rabbit | Multi Sciences (Hangzhou, China) | 1:1000 | ND |
| STAR | mouse | Santa Cruz (Santa Cruz, CA) | 1:1000 | ND |
| HSD11B1 | rabbit | Abcam (San Francisco, CA) | 1:1000 | 1:200 |
| CYP11A1 | rabbit | Cell Signaling Technology (Danvers, MA) | 1:1000 | 1:200 |
| NR5A1 | mouse | Santa Cruz (Santa Cruz, CA) | 1:1000 | ND |
| pAKT1 | rabbit | Abcam (San Francisco, CA) | 1:1000 | ND |
| AKT1 | rabbit | Abcam (San Francisco, CA) | 1:1000 | ND |
| pMTOR | rabbit | Cell Signaling Technology (Danvers, MA) | 1:1000 | ND |
| MTOR | rabbit | Cell Signaling Technology (Danvers, MA) | 1:1000 | ND |
| p4EBP-1 | rabbit | Cell Signaling Technology (Danvers, MA) | 1:1000 | ND |
| 4EBP-1 | rabbit | Cell Signaling Technology (Danvers, MA) | 1:1000 | ND |
| ATP5O | mouse | Abcam (San Francisco, CA) | 1:2000 | ND |
| SOX9 | rabbit | Abcam (San Francisco, CA) | 1:1000 | 1:500 |
| INSL3 | rabbit | Abcam (San Francisco, CA) | 1:1000 | ND |
| TRKC | rabbit | Cell Signaling Technology (Danvers, MA) | 1:1000 | ND |
| ACTB | rabbit | Beyotime Biotechnology (Shanghai, China) | 1:1000 | ND |

ND = Not detected; WB = Western blot; HS = Histochemical staining.
