# Supplementary material for: Neurotrophin‐3 stimulates stem Leydig cell proliferation during regeneration in rats
Source: J Cell Mol Med. 2020 Oct 22;24(23):13679–89. doi: 10.1111/jcmm.15886 (PMC7753877; doi:10.1111/jcmm.15886)
Supplement: Supplementary file 7 — SupInfoS5 [file JCMM-24-13679-s007.docx]

**Supplementary Table S5. General parameters of rats after treatment of NT-3**

| Parameters | Dosages（ng/testis） |  |  |
| --- | --- | --- | --- |
|  | 0 | 10 | 100 |
| **Body weight** |  |  |  |
| Before NT-3 treatment | 283.0±3.541 | 284.9±4.849 | 285.4±3.284 |
| After NT-3 treatment | 350.9±5.002 | 336.4±7.068 | 354.1±4.458 |
| **Testes weight** |  |  |  |
| After NT-3 treatment | 3.238±0.190 | 2.613±0.233 | 2.825±0.181 |
| **Relative testes** |  |  |  |
| Testes/Body weight | 0.00924±0.000572 | 0.00777±0.000675 | 0.00796±0.000451 |

Mean±SEM, n=8. * P < 0.05, ** P < 0.01, *** P < 0.001 indicate significant difference when compared to control (0 ng/testis) at each time point.
